# Esophageal Mucosal Permeability as a Surrogate Measure of Cure in Eosinophilic Esophagitis

**DOI:** 10.3390/jcm11144246

**Published:** 2022-07-21

**Authors:** Junji Chen, Tadayuki Oshima, Xinyi Huang, Toshihiko Tomita, Hirokazu Fukui, Hiroto Miwa

**Affiliations:** Division of Gastroenterology and Hepatology, Department of Internal Medicine, Hyogo Medical University, Nishinomiya 663-8501, Japan; ds2002@hyo-med.ac.jp (J.C.); huangxyeva@163.com (X.H.); tomita@hyo-med.ac.jp (T.T.); hfukui@hyo-med.ac.jp (H.F.); miwahgi@hyo-med.ac.jp (H.M.)

**Keywords:** eosinophilic esophagitis, IgG4, permeability, eotaxin-3, mast cell

## Abstract

This study aimed to evaluate the relationship of esophageal epithelial permeability with mast cell infiltration and IgG4 deposits as well as chemokine levels in eosinophilic esophagitis (EoE) patients before and after treatment. Biopsies from controls and EoE patients before and after treatment were analyzed. Hematoxylin and eosin staining was used to show eosinophil infiltration. Paracellular permeability of the esophageal epithelium was assessed using surface biotinylation. Immunohistochemical staining was performed to examine mast cell infiltration and IgG4 deposits. Gene expression of chemokines was evaluated by qRT-PCR. Esophageal epithelial infiltration of mast cells, IgG4 deposits, and permeability were significantly increased in EoE patients. Levels of interleukin-13, calpain-14, and eotaxin-3 mRNAs were significantly upregulated, while filaggrin, serine peptidase inhibitor Kazal type 7 (SPINK7), and involucrin mRNAs were significantly downregulated in EoE patients. In patients achieving histologic remission diagnosed by eosinophil counts, a subset of EoE patients with unchanged permeability after treatment showed increases in mast cell infiltration, IgG4 deposits, and interleukin-13, calpain-14, filaggrin, and SPINK7 expression, with decreased eotaxin-3 and involucrin. Other EoE patients with decreased permeability displayed decreased eotaxin-3, involucrin, and mast cell infiltration, no IgG4 deposits, and increased IL-13, calpain-14, filaggrin, and SPINK7. Increased permeability of the esophagus in EoE patients without eosinophil infiltration after treatment was associated with mast cell infiltration and IgG4 deposits.

## 1. Introduction

Eosinophilic esophagitis (EoE) is defined as a chronic inflammatory disorder that is immune- or food antigen-driven, clinically characterized by symptoms of dysphagia and eosinophil-predominant inflammation on esophageal biopsy [1,2]. As chronic inflammation with a very low spontaneous remission rate is characteristic of EoE, most patients require long-term therapy [3,4].

Esophageal remodeling and stricture develop either without treatment or with treatment under refractory conditions [5,6]. However, optimal histologic and endoscopic endpoints of therapy remain unclear. Peak eosinophil count is traditionally used to assess histologic activity in EoE [7]. Even when infiltration of few eosinophils is seen on esophageal biopsies after treatment, 53% of those EoE patients relapse after cessation of therapies [4].

The architecture of the esophageal epithelium is disrupted in EoE, accompanied by basal zone hyperplasia [8]. Our previous study showed that esophageal barrier function is decreased in patients with EoE [9]. Increased permeability of the esophageal mucosa could play an important role in the presentation of allergens to the immune system in EoE [10,11,12]. Mast cells are tissue-resident immune cells and are present in increased numbers in the epithelial layers of the esophagus in EoE, even under conditions of eosinophil elimination [13,14,15]. Furthermore, previous studies have detected immunoglobulin (Ig)G4 deposits in the esophageal epithelial tissues of EoE patients [16,17]. Immunohistochemical staining for mast cells and IgG4 can be useful for the diagnosis of EoE and also in determining the appropriate treatment [18].

Th2-type cytokines such as interleukin (IL)-13 are increased in allergen-exposed tissues and are seen at high levels in EoE [19,20]. Genome-wide association studies have revealed that alterations to the chemokine milieu are related to EoE [20,21]. Eotaxin-3 produced by epithelial cells plays a major role in attracting eosinophils to the esophagus [22]. Overexpression of calpain-14 (CAPN14) in esophageal epithelial cells results in a substantially impaired barrier function [23]. Downregulation of filaggrin (FLG) and involucrin (IVL) is associated with barrier dysfunction [24]. Loss of serine peptidase inhibitor Kazal type 7 (SPINK7) is thought to be the trigger in the pathogenesis of EoE [25].

The hypothesis of this study was that esophageal mucosal permeability could be related to histologic changes in EoE patients before and after treatment, as measured by mast cell infiltration and IgG4 deposits. This study therefore examined histologic findings as well as chemokine expressions in EoE patients before and after treatment, to better characterize the relationships between esophageal mucosal permeability and histologic changes in EoE.

## 2. Materials and Methods

### 2.1. Human Endoscopic Biopsies

Patients showing at least one esophageal symptom (such as odynophagia, heartburn/regurgitation, vomiting, dysphagia, nausea, and food impactions) and a peak eosinophil count ≥ 15 per high-power field (HPF) on esophageal biopsy without any treatment were diagnosed with EoE [1,2]. Patients under treatment were receiving a regular potassium-competitive acid blocker (P-CAB)/proton pump inhibitor (PPI) with or without topical corticosteroid treatment. Esophageal biopsies were taken before and after treatment. At least one follow-up endoscopy was performed for each patient. Histologic remission after treatment was defined as <15 eosinophils/HPF [26,27]. Controls were defined as patients who underwent endoscopy for clinical complaints but had 0 eosinophils/HPF and no histologic or endoscopic evidence of EoE, as previously reported [15]. All EoE patients and controls were recruited at Hyogo College of Medicine, Japan, between 2018 and 2020. We used a previously described method to deal with those samples [9]. Briefly, for each patient/control, two separate samples were obtained from each of the middle and distal esophagus. One of the two biopsy samples from the same region was immediately stored in RNAlater (Qiagen, Hilden, Germany) at −20 °C until measurement of messenger RNA (mRNA) levels. A second biopsy sample was used for the biotinylation assay and hematoxylin and eosin (HE), immunofluorescent, and immunohistochemical staining. This study was performed in accordance with the Declaration of Helsinki and was approved by the Ethics Committee/Institutional Review Board of Hyogo College of Medicine, Japan (No. 174). Each patient/control provided written informed consent.

### 2.2. Barrier Function Assays

The tight junction (TJ) permeability assay using surface biotinylation was performed according to previously described methods [9]. Briefly, esophageal biopsies were incubated with 1 mg/mL of EZ-Link Sulfo-NHS-LC-biotin (557 Da; Thermo Fisher Scientific, Waltham, MA, USA) in N-2-hydroxyethylpiperazine-N′-2-ethanesulfonic acid (HEPES)-buffered saline containing 1 mM of CaCl_2_ and 1 mM of MgCl_2_ for 30 min at room temperature. After washing three times with phosphate-buffered saline, samples were fixed overnight in 10% formalin at 4 °C, embedded in paraffin, and sectioned at a thickness of 4 μm. Sections were deparaffinized and washed three times with phosphate buffered saline, then incubated for 30 min with streptavidin (Texas red conjugate, Thermo Fisher Scientific) and 4′,6-diamidino-2-phenylindole (DAPI) (Thermo Fisher Scientific). Slides were examined under a confocal laser-scanning microscope (LSM780; Carl Zeiss, Oberkochen, Germany). The rate of biotinylated layer from the apical side in epithelial layers was calculated.

### 2.3. Quantitative Reverse Transcription Polymerase Chain Reaction (qRT-PCR)

Total mRNA was extracted using Trizol reagent (Thermo Fisher Scientific), according to the manufacturer’s instructions. Synthesis of cDNA was performed using a high-capacity cDNA reverse transcription kit (Applied Biosystems, Foster City, CA, USA). A PCR master mix and a QuantStudio^TM^ 12K Flex real-time PCR system (Applied Biosystems) were used for qRT-PCR.

TaqMan^®^ probes and primers for CAPN14 (Accession No. Hs00871882_m1; Applied Biosystems), SPINK7 (Hs00261445_m1), FLG (Hs00856927_g1), IVL (Hs00846307_s1), IL-13 (Hs00174379_m1), and eotaxin-3 (CCL26) (Hs00171146_m1) were Thermo Fisher Assay-On Demand gene expression products. The glyceraldehyde 3-phosphate dehydrogenase-encoding (GAPDH) gene (Hs 02758991_g1) was used as an endogenous control. The thermal cycler conditions were as follows: 2 min at 50 °C, 10 min at 95 °C, and 40 cycles of 15 s at 95 °C for denaturing and 1 min at 60 °C for annealing/extension. Amplification data were analyzed using QuantStudio^TM^ 12K Flex software version 1.3 (Applied Biosystems). The ΔΔCT method recommended by the manufacturer was used to compare relative expression levels.

### 2.4. Immunohistochemical and Immunofluorescent Staining

Paraffin-embedded, 4 µm-thick sections of biopsy specimens were deparaffinized as mentioned above. After antigen retrieval by autoclaving in tris-EDTA-based buffer (S2375; Dako, Carpinteria, CA, USA), non-specific binding was blocked with 3% hydrogen peroxide and protein blocking solution (X0909; Dako) for immunohistochemical staining. Mast cells were stained with rabbit anti-human CD117, c-kit (1:500; A4502, Dako) for 30 min at room temperature. Specimens were then sequentially incubated with secondary EnVision Dual Link System-HRP^®^ (K4063; Dako) for 30 min at room temperature and the chromogen diaminobenzidine, then counterstained with Mayer’s hematoxylin. Mast cells were counted in at least three representative non-overlapping HPFs at 400× magnification in a blinded manner.

Non-specific binding was blocked with 5% bovine serum albumin for immunofluorescent staining. IgG4 was stained with rabbit anti-human IgG4 antibody (1:1000; AB109493, Abcam, Cambridge, UK) for 60 min at room temperature. Specimens were then incubated with Cy3-conjugated goat anti-rabbit IgG (A120-201C3; Bethyl Laboratories, Montgomery, TX, USA) and DAPI for 30 min at room temperature. Slides were examined under a confocal laser-scanning microscope (LSM780). IgG4 deposits were defined as the existence of granular intercellular staining in the esophageal epithelia [17].

### 2.5. Statistical Analysis

All values are presented as the mean ± standard deviation (SD). Data were analyzed using unpaired *t*-tests (two-tailed) to compare controls and EoE patients before treatment, and paired *t*-tests (two-tailed) to compare EoE patients before and after treatment. The chi-square test was used to compare sex between controls and EoE patients. Fisher’s exact test was used to compare positive immunofluorescence between different groups. Significance was accepted at the level of *p* < 0.05. Analyses were performed using GraphPad Prism9 (GraphPad Software, San Diego, CA, USA).

## 3. Results

### 3.1. Subjects

A total of 22 subjects (11 EoE patients, 11 controls) met the inclusion criteria for the present study. No significant differences in age, sex, or body mass index were observed between controls and EoE patients (Table 1 and Table S1). None of the EoE subjects were diagnosed with allergy.

### 3.2. Eosinophil Infiltration and Permeability Changes in Controls and EoE Patients before and after Treatment

**Figure 1:**
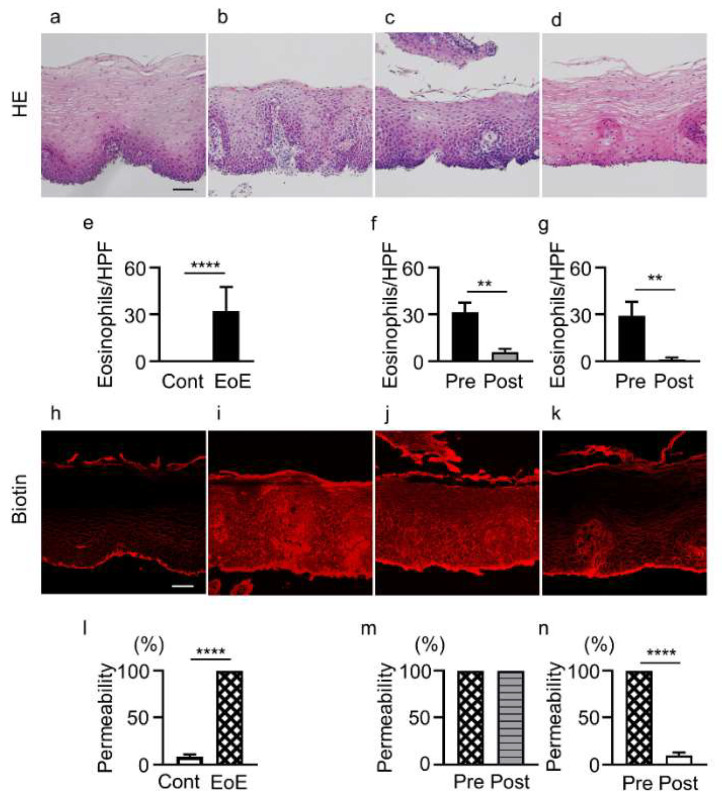
Infiltration of eosinophils and epithelial permeability in controls and EoE patients before and after treatment. (**a**–**d**) HE staining of biopsy sections. (**e**–**g**) Eosinophil counts in: controls (cont, *n* = 11) and EoE (*n* = 11, (**e**)), EoE before treatment (pre, *n* = 5) and after treatment (post, *n* = 5) among patients with unchanged permeability (**f**), and EoE before treatment (pre, *n* = 5) and after treatment (post, *n* = 5) among patients with decreased permeability (**g**). (**h**–**k**) Sections stained with biotin (red). (**l**) Percentage of biotinylation of controls (*n* = 11) and EoE before treatment (*n* = 11). (**m**,**n**) Esophageal epithelial permeability of EoE patients with unchanged permeability (*n* = 5, (**m**)) and decreased permeability (*n* = 5, (**n**)) after treatment. Representative HE and biotin staining of samples from controls (**a**,**h**), EoE patients before treatment (**b**,**i**), EoE patients with unchanged permeability after treatment (**c**,**j**), and EoE patients with decreased permeability after treatment (**d,k**). Data are expressed as mean ± standard deviation. EoE, eosinophilic esophagitis; HE, hematoxylin and eosin; HPF, high-power field. ** *p* < 0.01, **** *p* < 0.0001. Bar = 50 μm.

Eosinophil counts were increased in esophageal epithelial layers of EoE patients compared with controls (32.2 ± 15.4 vs. 0 eosinophils/HPF, *p* < 0.0001) (Figure 1a,b,e). The biotinylation reagent diffused through whole cell layers in samples from EoE patients, but was abruptly stopped at superficial layers in controls (100% vs. 8.3% ± 2.4%, *p* < 0.0001) (Figure 1h,i,l).

Among the 11 EoE patients, 4 patients (3 with P-CAB, 1 with PPI) did not achieve histologic remission after treatment (≥15 eosinophils/HPF). In 5 patients (3 with P-CAB, 2 with P-CAB and swallowed topical corticosteroid for 5.2 ± 2.3 months) achieving histologic remission after treatment (<15 eosinophils/HPF), eosinophil count was significantly decreased in esophageal epithelial layers (5.8 ± 2.2 vs. 31.4 ± 6.2 eosinophils/HPF, *p* < 0.01) (Figure 1b,c,f), while esophageal epithelial permeability was not decreased compared with that before treatment (100% vs. 100%) (Figure 1i,j,m).

In contrast, in 5 EoE patients achieving histologic remission (<15 eosinophils/HPF) after treatment (4 with P-CAB, 1 with P-CAB and swallowed topical corticosteroid for 11.0 ± 8.5 months), eosinophil count was significantly decreased (1.0 ± 1.4 vs. 29.2 ± 8.8 eosinophils/HPF, *p* < 0.01) (Figure 1b,d,g) and permeability was also significantly decreased compared with that before treatment (9.8% ± 3.1% vs. 100%, *p* < 0.0001) (Figure 1i,k,n). Three of the five patients showed no change in increased permeability after treatment even after histologic remission when the first follow-up endoscopy was performed, but showed decreased permeability when the second follow-up endoscopy was performed (Table S2).

### 3.3. Mast Cell Infiltration and IgG4 Deposits in Controls and EoE Patients before and after Treatment

**Figure 2:**
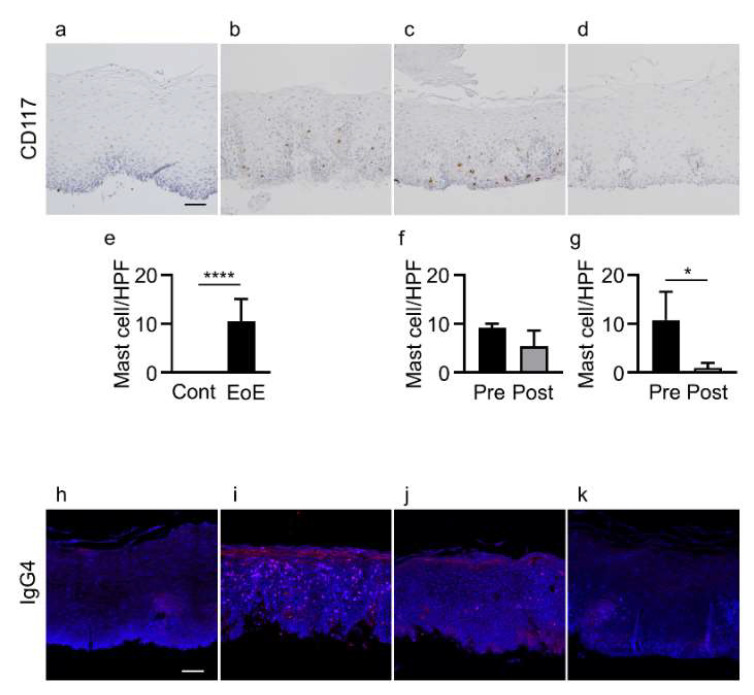
Infiltration of mast cells and IgG4 deposits in controls and EoE patients before and after treatment. (**a**–**d**) CD117 staining of esophageal biopsy sections. (**e**–**g**) Mast cell counts in: controls (cont, *n* = 11) and EoE (*n* = 11, (**e**)), EoE before treatment (pre, *n* = 5) and after treatment (post, *n* = 5) among patients with unchanged permeability (**f**), and EoE before treatment (pre, *n* = 5) and after treatment (post, *n* = 5) among patients with decreased permeability (**g**). (**h**–**k**) IgG4 immunofluorescence (red) and nuclear staining with DAPI (blue) in biopsy sections. Representative immunohistochemical and immunofluorescence staining of controls (**a**,**h**), EoE patients before treatment (**b**,**i**), EoE patients after treatment with unchanged permeability (**c**,**j**), and EoE patients after treatment with decreased permeability (**d**,**k**). Data are expressed as mean ± standard deviation. DAPI, 4′,6-diamidino-2-phenylindole; EoE, eosinophilic esophagitis; HPF, high-power field; Ig, immunoglobulin. * *p* < 0.05, **** *p* < 0.0001. Bar = 50 μm.

Mast cell counts in esophageal epithelial layers were significantly increased in patients with EoE compared with controls (10.6 ± 4.5 vs. 0/HPF, *p* < 0.0001) (Figure 2a,b,e). Mast cell counts in the 5 EoE patients who achieved histologic remission (<15 eosinophils/HPF) with unchanged esophageal epithelial permeability after treatment were not significantly decreased compared with before treatment (5.4 ± 3.2 vs. 9.2 ± 0.8/HPF) (Figure 2b,c,f). In contrast, mast cell counts were significantly decreased in the 5 EoE patients who achieved histologic remission with decreased permeability after treatment compared with those before treatment (1.0 ± 1.0 vs. 10.8 ± 5.7/HPF, *p* < 0.05) (Figure 2b,d,g).

IgG4 deposits were not detected in esophageal epithelial layers in any of the 11 controls, while IgG4 deposits were detected in all 11 EoE patients (*p* < 0.0001) (Figure 2h,i). In the group achieving histologic remission (<15 eosinophils/HPF) with unchanged permeability, IgG4 deposits were detected in 4 of the 5 EoE patients after treatment compared to all 5 EoE patients before treatment (Figure 2i,j). In contrast, in the group achieving histologic remission with decreased permeability, IgG4 deposits were not detected in any of the 5 EoE patients after treatment compared to all 5 EoE patients before treatment (*p* < 0.01) (Figure 2i,k) (Table S2).

### 3.4. Gene Expression in Controls and EoE Patients before and after Treatment

Levels of IL-13, CAPN14, and CCL26 mRNA in esophageal epithelial layers of EoE patients were significantly upregulated compared to controls. In contrast, levels of FLG, SPINK7, and IVL mRNA were significantly downregulated in EoE patients compared to controls (Table 2).

IL-13 and CAPN14 mRNA levels were not decreased after treatment in EoE patients achieving histologic remission (<15 eosinophils/HPF), with unchanged or decreased permeability compared to those before treatment. However, CCL26 mRNA levels were significantly decreased after treatment in EoE patients achieving histologic remission. In contrast, levels of FLG and SPINK7 mRNA, but not IVL mRNA, were significantly increased after treatment in patients achieving histologic remission (Table 2).

## 4. Discussion

In the present study, a subset of EoE patients after treatment displayed unchanged esophageal epithelial permeability without eosinophil infiltration and with infiltration of mast cells and IgG4 deposits. These data indicate that esophageal permeability could remain increased without eosinophil infiltration in epithelial layers. Assessing eosinophil infiltration alone may thus be insufficient to evaluate the efficacy of EoE treatments.

A peak count of 15 eosinophils/HPF is considered the minimum threshold for the diagnosis of EoE, and this cut-off has been used to predict endoscopic and histologic improvement of EoE after receiving treatment [28]. However, esophageal and abdominal symptoms might persist even after achieving such conditions of histologic remission [29]. When TJs are intact, biotin reagents cannot penetrate whole epithelial layers. However, when TJ structures and functions are disrupted, biotin molecules pass into the intercellular space [30]. In the present study, patients with EoE before treatment showed increased permeability compared to controls, consistent with previous findings [9,10]. Increased permeability could play an important role in the presentation of allergens to the immune system in EoE [11]. Similarly, in other atopic diseases (such as asthma and atopic dermatitis), high epithelial permeability has been considered to facilitate allergen sensitization [31,32]. Interestingly, a subset of EoE patients showing histologic remission after treatment still exhibited increased permeability in the present study, while other EoE patients showed decreased permeability after treatment. Eosinophil infiltration might recover before epithelial barrier functions recover. Thus, not only infiltrated eosinophil count in esophageal epithelial layers, but also recovery of esophageal epithelial permeability, may need to be considered as the endpoints of therapy.

Mast cells in the esophageal epithelial layers of EoE patients release mediators that may be involved in the remodeling of those layers [13,15]. Mast cell infiltration in EoE patients before treatment was associated with increased permeability in the present study. A previous report showed that mast cell infiltration was associated with histologic basal zone hyperplasia in pediatric patients with EoE, even in the absence of evidence of eosinophil infiltration [33]. In the present study, a subset of EoE patients showed increased permeability with mast cell infiltration without eosinophil infiltration even after treatment. In contrast, infiltration of mast cells ceased when esophageal permeability decreased after treatment. Similar associations between mast cells and furrows have been found in adult and pediatric EoE patients [33,34]. The longer lifespan of mast cells compared with eosinophils might contribute to persistent infiltration of mast cells even in the absence of eosinophil infiltration [35].

IgG4 levels in esophageal tissue are elevated in EoE patients and IgG4-positive plasma cells have been found in esophageal epithelial layers [16,17]. Similarly, IgG4 deposits were detected in EoE patients in the present study. Interestingly, IgG4 deposits were still detected in those EoE patients showing unchanged epithelial permeability after treatment. In contrast, when esophageal epithelial permeability decreased, IgG4 deposits were no longer detected. These findings suggest that IgG4 deposits in epithelial layers might correlate with increased epithelial permeability. Further studies are needed to clarify the correlation between IgG4 and permeability.

In IgG4-related diseases, IL-13-positive mast cells are increased [36], and IgE-positive mast cells, rather than T cells, release cytokines such as IL-4 and IL-10 [37]. In the present study, when mast cell infiltration was evident in the esophageal epithelial layers of EoE patients after treatment, IgG4 deposits were detected. In contrast, when mast cell infiltration ceased, IgG4 deposits were no longer detected. Previous studies have indicated that IgG4 antibody plays a protective role by inhibiting IgE activity to prevent mast cell degranulation [38,39]. Further investigations are warranted to clarify whether IgG4 is protective against mast cell activation in EoE.

Levels of IL-13, eotaixin-3, and CAPN14 mRNAs in esophageal mucosa were upregulated in EoE patients before treatment, while FLG and IVL mRNA levels were downregulated in the present study. These data are consistent with findings from previous studies [40,41]. SPINK7, an esophageal-enriched protein, inhibits serine protease kallikrein 5 from activating protease-activated receptor 2, leading to cytokine production, including thymic stromal lymphopoietin that promotes type 2 immune responses [42]. As might be expected, levels of SPINK7 mRNA were downregulated in EoE patients before treatment in the present study. Interestingly, levels of FLG and SPINK7 mRNA were increased, while eotaxin-3 levels were decreased after treatment, even when esophageal epithelial permeability was not decreased.

This study showed several limitations. First, the sample size was small due to the low incidence of EoE in Asia [43]. However, our findings were generally consistent with previous data. Esophageal epithelial infiltration of mast cells, IgG4 deposits, and permeability were significantly increased in EoE patients [9,14,17]. Levels of IL-13, CAPN14, and eotaxin-3 mRNAs were significantly upregulated, while FLG, SPINK7, and IVL mRNAs were significantly downregulated in EoE patients [9,20,21]. Moreover, some previous comparable studies were of a similar size due to the limited number of patients [12,22]. Second, although we identified differences in recovery timing among infiltration of eosinophils and mast cells, epithelial permeability, and IgG4 deposition, these findings do not reflect how infiltration of mast cells and IgG4 deposits recover according to epithelial permeability. Third, our biopsy samples were taken from two positions in the esophagus, which was less than recommended in the guideline [28].

## 5. Conclusions

In summary, esophageal permeability was increased in EoE patients. After treatment, a subset of EoE patients showed esophageal epithelial disruption, residual mast cell infiltration, and IgG4 deposits without eosinophil infiltration in the esophageal epithelia. Increased epithelial permeability in EoE might correlate with mast cell infiltration and IgG4 deposition.

## Figures and Tables

**Table 1:** Characteristics of controls and EoE patients.

	Control (*n* = 11)	EoE (*n* = 11)	*p*-Value
Age (years, mean ± SD)	53.3 ± 12.2	43.4 ± 12.9	0.08
Sex (male/female)	5/6	6/5	0.67
BMI (kg/m^2^, mean ± SD)	24.0 ± 4.8	23.0 ± 4.3	0.61

Abbreviation: BMI, body mass index; EoE, eosinophilic esophagitis; SD, standard deviation.

**Table 2:** Gene expression in biopsy samples from controls and EoE patients before and after treatment.

	Control (*n* = 11)	EoE (*n* = 11)	*p*-Value	Increased Permeability ^§^ (*n* = 5)		*p*-Value	Decreased Permeability ^§§^ (*n* = 5)		*p*-Value
				Pre	Post		Pre	Post	
IL-13	0.07 ± 0.12	10.37 ± 6.82	<0.0001	9.37 ± 5.73	2.42 ± 2.81	0.06	8.19 ± 7.80	0.57 ± 1.12	0.07
CAPN14	1.00 ± 0.58	7.56 ± 5.75	0.001	5.78 ± 3.86	4.06 ± 4.01	0.09	8.82 ± 7.46	3.96 ± 3.33	0.08
CCL26	0.30 ± 0.38	108.40 ± 93.14	0.001	81.96 ± 48.20	12.56 ± 17.06	0.03	61.51 ± 46.09	20.40 ± 42.68	0.03
FLG	1.48 ± 1.42	0.02 ± 0.03	0.007	0.23 ± 0.47	0.80 ± 0.38	0.007	0.03 ± 0.04	0.59 ± 0.16	0.001
SPINK7	0.33 ± 0.22	0.05 ± 0.06	0.0004	0.05 ± 0.07	0.30 ± 0.18	0.02	0.02 ± 0.01	0.27 ± 0.96	0.005
IVL	0.06 ± 0.03	0.02 ± 0.02	0.003	0.03 ± 0.02	0.04 ± 0.03	0.32	0.02 ± 0.02	0.03 ± 0.02	0.07

All data were expressed as mean ± standard deviation. ^§^ EoE patients after treatment achieving histologic remission with increased permeability, ^§§^ EoE patients after treatment achieving histologic remission with decreased permeability. Abbreviation: CAPN14, calpain-14; CCL26, eotaxin-3; EoE, eosinophilic esophagitis; FLG, filaggrin; IL, interleukin; IVL, involucrin; SPINK7, serine peptidase inhibitor Kazal type 7.

## Data Availability

The data that support the findings of this study are available upon request from the corresponding author. The data are not publicly available due to privacy or ethical restrictions.

## Supplementary Materials

The following supporting information can be downloaded at: https://www.mdpi.com/article/10.3390/jcm11144246/s1, Table S1. Demographics, symptoms, and endoscopic and histologic findings of control subjects and EoE patients before treatment. Table S2. Treatment, symptoms, endoscopic and histologic findings of EoE patients after treatment.
