# Supplementary material for: Esophageal Mucosal Permeability as a Surrogate Measure of Cure in Eosinophilic Esophagitis
Source: J Clin Med. 2022 Jul 21;11(14):4246. doi: 10.3390/jcm11144246 (PMC9319696; doi:10.3390/jcm11144246)
Supplement: Supplementary file 1 [file jcm-11-04246-s001.zip › jcm-1803626-supplementary.pdf]

Table S1. Demographics, symptoms, and endoscopic and histologic findings of control subjects and EoE patients before treatment.

	Age (y)	Sex	BMI	Symptoms	Endoscopic findings	Histological findings			
						Eosinophils/HPF	Permeability	Mast cells/HPF	IgG4 deposits
EoE patients									
1	41	Male	31.6	Odynophagia	Furrows, white exudates	38	High	10	+
2	64	Female	21.5	Dysphasia	Furrows, white exudates	35	High	10	+
3	45	Male	24.6	Odynophagia	Furrows	33	High	10	+
4	32	Female	19.1	Heartburn	Furrows, white exudates	22	High	10	+
5	51	Female	22.4	Odynophagia	Furrows	29	High	8	+
6	59	Male	27.1	Heartburn	Furrows, white exudates	23	High	4	+
7	54	Male	27.4	Dysphasia	Rings, furrows, white exudates	17	High	20	+
8	35	Male	20.8	Heartburn	Furrows, white exudates	69	High	12	+
9	20	Male	17.4	Heartburn	Rings, furrows, white exudates	48	High	17	+
10	41	Male	21.6	Nausea	Furrows, white exudates	20	High	8	+
11	35	Female	19.1	Dysphasia	Furrows	20	High	7	+
Controls									
12	65	Female	22.5	No	Normal	0	Normal	0	-
13	49	Female	17.7	No	Normal	0	Normal	0	-
14	69	Female	19.5	No	Normal	0	Normal	0	-
15	41	Male	19.8	No	Normal	0	Normal	0	-
16	45	Female	33.6	No	Normal	0	Normal	0	-
17	63	Female	28.9	No	Normal	0	Normal	0	-
18	61	Male	22.8	Heartburn	Normal	0	Normal	0	-
19	57	Male	25.9	No	Normal	0	Normal	0	-
20	57	Male	21.5	No	Normal	0	Normal	0	-
21	52	Male	28.4	No	Normal	0	Normal	0	-
22	27	Female	22.9	No	Normal	0	Normal	0	-

Abbreviation: BMI, body mass index; EoE, eosinophilic esophagitis, HPF, high-power field.

Table S2. Treatment, symptoms, endoscopic and histologic findings of EoE patients after treatment.

	Medication (months)	Symptoms	Endoscopic findings	Histologic findings			
				Eosinophils/HPF	Permeability	Mast cells/HPF	IgG4 deposits
First follow-up							
1	Vonoprazan 20 mg + topical steroid (5)	No symptoms	Shallow furrows	5	High	1	-
2	Vonoprazan 20 mg (3)	No symptoms	Shallow furrows	3	high	3	+
3	Vonoprazan 10 mg (9)	No symptoms	Furrows, white exudates	8	High	7	+
4	Vonoprazan 20 mg + topical steroid (7)	No symptoms	Shallow furrows	8	High	7	+
5	Vonoprazan 20 mg (9)	Odynophagia	Furrows	5	High	8	+
6	Vonoprazan 20 mg + topical steroid (4)	Heartburn	Shallow furrows	3	Decreased	2	-
7	Vonoprazan 20 mg (3)	Dysphasia	Furrows	2	Decreased	2	-
8	Vonoprazan 20 mg (28)	Heartburn	Furrows	15	High	13	+
9	Vonoprazan 10 mg (3)	No symptoms	Rings	72	High	8	+
10	Vonoprazan 20 mg + topical steroid (11)	Nausea	furrows, white exudates	20	High	5	+
11	Esomeprazole 20mg (2)	Dysphasia	Furrows	22	High	9	+
Second follow-up							
1	Vonoprazan 20 mg (11)	No symptoms	Normal	0	Decreased	1	-
2	Vonoprazan 10 mg (6)	No symptoms	Normal	0	Decreased	0	-
3	Vonoprazan 10 mg (14)	No symptoms	Normal	0	Decreased	0	-

Abbreviation: HPF, high-power field
